# Production and Potential Applications of Bioconversion of Chitin and Protein-Containing Fishery Byproducts into Prodigiosin: A Review

**DOI:** 10.3390/molecules25122744

**Published:** 2020-06-13

**Authors:** San-Lang Wang, Van Bon Nguyen, Chien Thang Doan, Thi Ngoc Tran, Minh Trung Nguyen, Anh Dzung Nguyen

**Affiliations:** 1 Department of Chemistry, Tamkang University, New Taipei City 25137, Taiwan; doanthng@gmail.com (C.T.D.); tranngoctnu@gmail.com (T.N.T.); 2 Life Science Development Center, Tamkang University, New Taipei City 25137, Taiwan; 3 Institute of Research and Development, Duy Tan University, Danang 550000, Vietnam; 4 Department of Science and Technology, Tay Nguyen University, Buon Ma Thuot 630000, Vietnam; nguyenminhtrung2389@gmail.com; 5 Institute of Biotechnology and Environment, Tay Nguyen University, Buon Ma Thuot 630000, Vietnam; nadzungtaynguyenuni@yahoo.com.vn

**Keywords:** prodigiosin, *Serratia marcescens*, marine drugs, chitin, bioconversion

## Abstract

The technology of microbial conversion provides a potential way to exploit compounds of biotechnological potential. The red pigment prodigiosin (PG) and other PG-like pigments from bacteria, majorly from *Serratia marcescens*, have been reported as bioactive secondary metabolites that can be used in the broad fields of agriculture, fine chemicals, and pharmacy. Increasing PG productivity by investigating the culture conditions especially the inexpensive carbon and nitrogen (C/N) sources has become an important factor for large-scale production. Investigations into the bioactivities and applications of PG and its related compounds have also been given increased attention. To save production cost, chitin and protein-containing fishery byproducts have recently been investigated as the sole C/N source for the production of PG and chitinolytic/proteolytic enzymes. This strategy provides an environmentally-friendly selection using inexpensive C/N sources to produce a high yield of PG together with chitinolytic and proteolytic enzymes by *S. marcescens*. The review article will provide effective references for production, bioactivity, and application of *S. marcescens* PG in various fields such as biocontrol agents and potential pharmaceutical drugs.

## 1. Introduction

Chitin and its derivatives have numerous applications in the fields of environment protection, fine chemistry, and pharmacy [1,2,3,4,5,6,7,8,9,10,11]. Fishery processing byproducts, such as shrimp and crab shells, shrimp heads, and squid pens, are the main sources of chitin and chitosan which are conventionally prepared via chemical pretreatments of acid demineralization and hot-alkali deproteinization [1,5,9,11,12,13,14,15,16]. Chitin, chitosan, colloidal chitin, and water soluble chitosan have commonly been used as the major carbon/nitrogen (C/N) sources for the isolation of strains producing chitinolytic enzymes and the production of chitinolytic enzymes by these isolated strains [9,15,16,17,18]. To cut down fermentation expenses, the inexpensive materials of shrimp heads, shrimp shells, crab shells, and squid pens have recently been evaluated as the sole C/N sources for the production of bioactive compounds [5,6,9,12,13,14,15,16,19,20,21,22,23,24,25,26,27,28,29,30,31,32,33,34,35,36,37,38,39,40,41,42,43,44,45,46,47,48,49,50,51,52,53,54,55,56,57,58,59,60,61,62,63,64,65,66,67,68,69,70,71,72,73,74]. The recovery of chitin-containing fishery byproducts as the C/N source not only solves the environmental protection problem but also decreases the production costs of chitin and its derivatives.

Pigments of microbial origin have received much more attention in recent studies due to their versatile applications in agriculture, food, cosmetics, textile, and pharmacy. The antimicrobial, anti-cancer, and anti-parasite bioactivities confer the bacterial pigments with the potential to be developed as pharmaceutical products [75,76,77,78]. The worldwide concern recently has been to extend inclination towards the use of natural materials in place of artificial ones because of several advantages including concerns over environmentally-friendly sustenance [77,79]. Consequently, the uses of bacterial pigments have expanded much more than synthetic pigments [80]. Prodiginines (PGs), a family of red microbial pigments, have received much attention because of their considerable bioactivities [59,60,61,79,80,81,82,83,84,85,86,87,88,89,90,91,92,93,94,95,96,97,98,99,100,101,102,103,104,105,106,107,108,109,110,111,112,113,114,115,116,117,118,119,120,121,122,123,124,125,126,127,128,129,130,131,132,133,134,135,136,137,138,139,140,141,142,143,144,145,146,147,148,149,150], including antimicrobial [79,80,81,82,83,84,85,86,87,88,89,90,91,92,93,94,95,96,97], antiparasitic [102,103,104,105,106,107,108,109], insecticidal [60,61,110,111,112,113], anti-cancer [59,97,114,115,116,117,118,119,120,121,122,123,124,125,126,127,128,129,130,131,132,133,134,135,136,137,138,139,140,141,142,143,144,145,146,147], anti-oxidant [145,146], anti-inflammatory [145,146], immunosuppressant [147], and algicidal [99,148,149,150] activities. The potential applications of PGs as natural dyes have also been infrequently investigated for their use in textiles [101,151,152,153,154], candles [155], and cosmetics [96].

**Figure 1:**
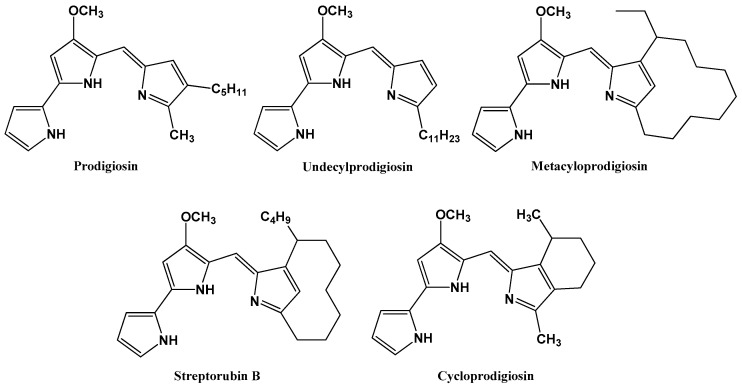
Structures of prodigiosin, undecylprodigiosin, metacyloprodigiosin, streptorubin B, and cycloprodigiosin.

PGs include prodigiosin (PG, 2-methyl-3-pentyl-6-methoxyprodiginine), undecylprodigiosin, metacycloprodigiosin, streptorubin B, and cycloprodigiosin [156,157,158,159] (Figure 1). Among PGs, PG is the first member which appears dark red and can be easily dissolved in organic solvents such as ethanol, methanol, and acetone [160,161,162]. The name of PG derived from Kraft in 1902. PG was first isolated in the 1920s, its structural features were identified in the 1930s, and structure elucidation was completed in the 1960s [163,164,165,166]. *Serratia marcescens* is the major source of PG production [59,60,61,62,81,82,83,84,85,86,87,88,89,90,91,92,93,94,95,96,97,98,99,100,101,102,103,104,105,106,107,108,109,110,111,112,113,114,115,116,117,118,119,120,121,122,123,124,125,126,127,128,129,130,131,132,133,134,135,136,137,138,139,140,141,142,143,144,145,146,147,148,149,150,151,152,153,154,155,156,157,158,159,160,161,162,163,164,165,166,167,168,169,170,171,172,173,174,175,176,177,178,179,180,181,182,183,184,185,186,187,188,189,190,191,192,193,194,195,196,197,198,199,200,201,202,203,204,205,206,207,208,209,210,211,212,213,214]. PGs are also produced by some other bacterial strains including *Serratia rubidaea* [154,177], *Alteromonas rubra* [178], *Janthinobacterium lividum* BR01 [179], *Rugamonas rubra* [180], *Streptomyces longisporus ruber* 100-19 [181], *Streptomyces coelicolor* [182], *Streptomyces spectabilis* BCC 4785 [105], *Streptomyces* fusant NRCF69 [91], *Streptomyces* sp. [106], *Vibrio* sp. C1-TDSG02-1 [116,118], *Vibrio* sp. KSJ45 [101], *V. gazogenes* [183], *V. psychroerythrus* [184], *Pseudomonas magnesiorubra* [185], *P. putida* KT2440 [188,189], *Streptoverticillium* sp. 26-1 [191], *Streptoverticillium rubrireticuli* [181], *Pseudoalteromonas* sp. [186], *Pseudoalteromonas rubra* [100], *Actinomycetes* [146,187], and a gene recombinant strain of *Pseudomonas putida* [185,189].

Some PG-related reviews reported the production of PG from *S. marcescens* using commercial media such as nutrient broth, peptone glycerol broth, and seed oils as the C/N sources [156,161,162,166,168,171]. The biological potentials of PG especially in the field of anticancer have also been reviewed [156,157,158,159,160,161,162,166,168,169,171]. For example, a review of the characteristics and potential therapeutic anticancer-drug applications of PG from *Serratia* was introduced by Darshan and Manonmani [180], while the structure, chemical synthesis, and biosynthesis of PGs as natural products were summarized by Hu et al. [157]. Additionally, synergistic inhibitory effects of chitinolytic enzymes [36,37,39,86,87,88,89,90], proteolytic enzymes [36,61,89,192,193,194,195], and *S. marcescens* PG were estimated for biocontrol in plant cultivation [85,86,87,88,89,90]. Biosurfactant produced by *S. marcescens* was also evaluated [167,196,197,198]. Recently, chitin and protein-containing marine byproducts have been utilized as the sole C/N source by *S. marcescens* TKU011 for the simultaneous production of PG [59,60,61,62], chitinolytic enzymes, and proteolytic enzymes [36,37]. The present review discusses the foremost accomplishments in the production and isolation of *S. marcescens* PG, particularly the application of shrimp heads, shrimp and crab shells, and squid pens as C/N sources for PG production by *S. marcescens* TKU011 [59,60,61,62]. Additionally, the uses of *S. marcescens* PG in several fields, especially as biocontrol agents, are also comprehensively reviewed.

## 2. Production of PGs

PG produced by *S. marcescens* has been considered as a promising aim for drug development due to the described antifungal, immunosuppressive, and antiproliferative bioactivities. Hence, the culture conditions for large-scale production to ameliorate PG productivity becomes an essential issue [75,76,77,78,147]. To gain higher PG productivity, important factors for evaluation include the media composition, inorganic phosphate availability, temperature, and pH [199,200]. In the case of C/N source, the most commonly used media for PG production were commercial media, such as nutrient broth and peptone glycerol broth. For example, Giri et al. found that the PG productivity by *S. marcescens* was 520 mg/L and 569 mg/L in nutrient broth (28 °C) and peptone glycerol broth (30 °C), respectively [201]. Furthermore, the supplement of fatty acid-containing plant oils was found to show a positive effect on PG productivity which increased to 16,680 mg/L in sesame seed broth (28 °C) [201]. Kamble and Hiwarale reported that *S. marcescens* showed better PG productivity in nutrient broth than in peptone glycerol broth [173]. Regarding the use of agricultural byproducts as C/N sources, ethanol and cassava wastewater were used for PG production by *S. marcescens* 389 [190], and *S. marcescens* UCP1549 [174], respectively. Further, chitin and protein-containing fishery byproducts have been utilized for PG production by *S. marcescens* TKU011 [59,60,61,62].

For the production of exopolysaccharides (EPS), *Paenibacillus polymyxa* EJS-3 has been reported to produce the highest EPS productivity (35.26 g/L) by using sucrose (188.2 g/L) and yeast extract (25.8 g/L) as the C/N source [56]. Recently, squid pens were utilized successfully to produce the highest yield of EPS from *Paenibacillus* sp. TKU023 (41.25 g/L) and *P. macerans* TKU029 (35.75 g/L) [56]. This inspired us to isolate the PG-producing strain, *S. marcescens* TKU011 using squid pens as the C/N source. The PG productivity of using chitin and casein, squid pens, shrimp shells, and shrimp heads as the sole C/N source by *S. marcescens* TKU011 was 4620 mg/L [59], 2480 mg/L, 190 mg/mL, and 30 mg/L, respectively [61].

Table 1 summarizes the comparison of the reported PG yield of *S. marcescens* by using different C/N sources. Chen et al. reported that a C/N ratio of 6/4 of starch/peptone (1.6% starch; 1.067% peptone) achieved a high PG yield (6700 mg/L) by *S. marcescens* C3. PG yield of 7070 mg/L was achieved after optimizing the concentrations of FeSO_4_·4H_2_O (0.56 mM) and MnSO_4_·4H_2_O (3.25 mM) [172]. Kamble and Hiwarale [173] studied *S. marcescens* PG production in peptone glycerol broth and nutrient broth and observed that the highest PG productivity was 1335 mg/L and 1845 mg/L, respectively after three days of cultivation [173]. Different from most other studies, the enhancing effects of oil supplements on PG production were not observed in this study [173]. The comparison of PG productivity by *S. marcescens* SR_1_ in nutrient broth and glycerol-yeast extract media was evaluated by Parani and Saha [81]. The results showed that a higher PG productivity (765 mg/L) may be achieved by supplementation of 4% vegetable oil mixture (sunflower, coconut, and olive oil) [81]. Medium containing ethanol (1.5%) and cottonseed meal (1.5%) omitted inorganic salts (phosphate and NaCl) and afforded *S. marcescens* S389 to produce up to 3000 mg/L PG [190]. Medium supplemented with powdered peanut, coconut, sesame, and castor seed was evaluated for PG production by *S. marcescens* PP1. The maximum yield of PG reached 1595 mg/L in powdered peanut-supplemented medium. Additionally, PG production by supplementing maltose and glucose to sunflower seed media reached 1556 mg/L and 1525 mg/L, respectively [82]. Peptone (1%) and maltose (0.5%) were used as the C/N source by *S. marcescenss* subsp. *lawsoniana* HDZK-BYSB107 for the production of PG (656 mg/L), which showed antibacterial and antitumor activities [143].

Cassava wastewater supplemented with 2% mannitol was used by *S. marcescens* UCP1549 to produce PG (49,500 mg/L) [174]. To avoid using individual fatty acid as a substrate for reducing the cost of industrial production, Giri et al. investigated the effect of different fatty acid-containing media for PG production and found that the higher yield was obtained in peanut seed medium (38,750 mg/L) and sesame seed broth (16,680 mg/L) [201]. Crude glycerol (a waste from biodiesel industry) was supplemented with peptone as a C/N source for PG production by *S. marcescens* MN5 [202]. Ram horn peptone (0.4%) supplemented in mannitol medium showed enhanced PG production (277.74 mg/L) by *S. marcescens* MO-1 [170]. To decrease the medium cost by using tannery fleshing (TF) as a C/N source, the proteinaceous byproducts of leather industries, Arivizhivendhan et al. studied the PG production in TF (30%, *w*/*w*) and wheat bran (70%, *w*/*w*)-containing solid media. The highest PG productivity reached was 70,402 mg per kg of TF [93]. The use of rice bran as a C/N source for *S. marcescens* PG production was reported by Arivizhivendhan et al., who showed that the PG produced showed antioxidant and antimicrobial activities against foodborne pathogens [92]. Natural substrates such as sweet potato, sesame, and mahua flower extract at different concentrations have also been used for PG production by *S. marcescens* [171]. The highest PG yield was 4800 mg/L at the final optimized composition of sweet potato powder/casein-containing medium. The purified PG showed antimicrobial [171] and nematicidal [111] activities.

*Janthinobacterium lividum* BR01 is a psychrotrophic strain that produces PG and heptyl prodigiosin when grown at cool temperatures. The gene cluster of the PG pathway was cloned from *J. lividium* BR01 and expressed in *Escherichia coli*, which showed differences in the responsible gene cluster of *Serratia* sp. [179]. Domröse et al. integrated the PG biosynthesis gene cluster of *S. marcescens* in *Pseudomonas putida* KT2440 to construct constitutive PG production strains [188]. The PG productivity obtained was 94 mg/L using the Terrific Broth medium [189]. The PGs productivity of *Streptomyces* fusant NRCF69, when peanut seed broth, sunflower oil broth, or dairy processing wastewater broth alone or supplemented with 0.5% mannitol were used as the C/N source, was 42,030 mg/L, 40,110 mg/L, 36,700 mg/L, and 47,000 mg/L, respectively [91].

The correlation between extracellular proteases synthesis and PG synthesis in *S. marcescens* VI was investigated and showed that chloramphenicol (an inhibitor of protein synthesis) inhibits the synthesis of both extracellular proteases and PG [182,183,184,185,186,187,188,189,190,191,192,193,194]. Similar results were also observed when using a mixture of 18 natural amino acids; asparagine and ammonium ions repressed the synthesis of both PG and protease [182,183,184,185,186,187,188,189,190,191,192,193,194]. On the contrary, leucine exhibited inducing effects on both the synthesis of exocellular protease and PG by *S. marcescens* VI [182,183,184,185,186,187,188,189,190,191,192,193,194]. The biosynthetic pathway for *Serratia* sp. producing PG involves separate pathways using different metabolites which then couple in the final condensation step [203]. The 5-methyl-4-pentyl-3,4-dihydro-2*H*-pyrrole and a transaminase have been shown as the intermediates involved in the PG biosynthesis by *Serratia* sp. ATCC 39006 [204] and involved in the biosynthesis of 2-methyl-3-*n*-amyl-pyrrole (MAP) from *Serratia* sp. FS14 [205], respectively.

Based on the hydrophobic properties of PG, efficient extraction procedures for PG purification from the culture supernatant of *P. putida* [188], *Serratia* sp. KH-95 [206], and *S. marcescens* SMDR [207] have been studied by adsorption to the materials with hydrophobic surfaces of polyurethane [188], internal adsorbent using acidified methanol and phase separation [206], and macroporous polymeric adsorption resin of Diaion HP-20 resins [207]. The transmembrane transport of PG producing *S. marcescens* ATCC 8100 and the permeability barrier of the cell membrane were studied by using a model membrane platform with a planar lipid bilayer [151]. The results showed that the mass transfer of the intracellular PG was affected by its size and surface electrical properties and therefore could be modulated by physical and chemical methods [151]. The immobilization strategy to increase PG production was investigated by Chen et al. [172]. The PG productivity of *S. marcescens* C3 enhanced seven-fold to 15,600 mg/L by using immobilized cells in calcium alginate beads. For increasing the recovery yield of PG, adsorption chromatography was studied to separate and purify PG directly. Wang et al. [208] reported that the use of 0.1% Tween 80 (a nonionic surfactant) may improve the release of PG from the cell envelope. The recovery of PG from the culture broth increased from 50% (using the conventional method) to 83% (with a high loading capacity of the adsorbent X-5 resin) [208]. To investigate the extraction methods which may purify PG with high yield and cost-effectivity, Khanam and Chandra tried six different extraction methods including homogenization, ultrasonication, freezing and thawing, heat treatment, organic solvents, and inorganic acids to evaluate the PG yield. The results showed that the highest amount of extraction was achieved by ultrasonication (98.1%) and the lowest by freezing and thawing (31.8%) methods [209].

The use of bioadsorbents for adsorption of PG was compared among *Lactobacillus paracasei* subsp. *paracasei* TKU012 cells, cicada casting, and four chitin-containing materials including squid pens, shrimp shells, α-chitin, and β-chitin [62]. The best result was observed in *L. paracasei* subsp. *paracasei* TKU012, followed by cicada casting, shrimp shells, squid pens, β-chitin, and α-chitin. The cells of lactic acid bacterium (strain TKU012) and cicada casting may have the potential to recover and purify PG from the PG-containing culture broth of *S. marcescens* TKU011 [62]. To increase *S. marcescens* TKU011 PG production in the medium containing squid pens, the effects of phosphate and ferrous ion supplementation, autoclave treatment, and aeration were studied [60]. The results showed that the 40-min autoclaved medium enhanced PG productivity 2.5-fold to 2480 mg/L [60]. Nguyen et al. further investigated PG production by *S. marcescens* TKU011 using 1% α-chitin and 0.6% casein as the C/N source and obtained the highest yield of 4620 mg/L. α-Chitin and CaSO_4_ were found to play an important role in enhancing PG production by *S. marcescens* [59].

Generally, peanut seed broth received remarkable PG productivity (47,000 mg/L) which was approximately 10-fold greater than those using chitin-containing fishery byproducts fermented by *S. marcescens* TKU011. Considering the utilization of obtaining culture broth for biological control, chitin and protein-containing byproducts (1–3 USD/kg) as the C/N source provide PG, and chitinolytic and proteolytic enzymes might also be expected to have potential applications.

## 3. Bioactivity and Application of PG

### 3.1. Antimicrobial Activity

The antifungal activity of PG produced from *S. marcescens* is investigated usually against fungal pathogens. The PG from *S. marcescens* SR_1_ showed the maximum inhibitory activity against the fungal pathogens *Helminthosporium sativum*, *Fusarium oxysporium*, and *Rhizoctonia solani*, in decreasing order [81]. Picha et al. reported that the chitin-supplemented agar medium used for the growth and production of PG by *S. marcescens* PP1 showed high inhibition to the tested fungal pathogens including *Alternaria alternata*, *Aspergillus niger*, and *F. oxysporum*. The tested fungal species of *A. niger*, *Mucor* sp., and *Rhizopus* sp. were resistant to the produced pigment with no clear zone of inhibition [82]. PG has been reported to display antagonistic effects on the tested fungal strains by increasing permeability in the fungal membrane. Among the tested fungi on potato dextrose agar (PDA) plates, *F. oxysporum* was highly inhibited by the PG treatment. PG enables *S. marcescens* D1 (an endofungal bacterium) to invade fungal hyphae and spread over the culture of *F. oxysporum* to result in mycelial death. [213].

In a 0.2% colloidal chitin supplemented Luria broth, *S. marcescens* B2 produced PG and four extracellular chitinolytic enzymes which showed antifungal activities against the phytopathogens *R. solani* (caused cyclamen damping-off and rice sheath blight), *F. oxysporum* (caused fusarium wilt), and *Botrytis cinerea* (caused gray mold) [85,86,87]. These four chitinolytic enzymes were detected among the extracellular proteins of *S. marcescens* B2. Among the four chitinolytic enzymes produced by *S. marcescens* B2, two enzymes showed an inhibitory effect against the spore germination of *B. cinerea*. The PG extracted and purified from the bacterial cells of *S. marcescens* B2 also showed inhibition against the spore germination of *B. cinerea*. A synergistic effect of the *S. marcescens* B2 produced PG and chitinolytic enzymes was also observed against the tested fungal pathogens [85,86,87]. Gutiérrez-Román et al. [88] also reported that the combination of *S. marcescens* CFFSUR-B2 produced PG and chitinolytic enzymes showed synergistic inhibitory effect on the germination and germ tube growth of *Mycosphaerella fijiensis* ascospores. Based on the results of toxic effects similar to that of benzimidazole on ascospore germination, the authors suggested that the combination of PG and chitinolytic enzymes may have potential use in the biocontrol of black Sigatoka disease caused by *Mycosphaerella fijiensis* [88]. *S. marcescens* ETR17 produced PG, several hydrolytic enzymes (chitinase, protease, cellulase, and lipase), and plant growth-enhancing compounds (iodoacetic acid and siderophore). This strain was isolated as a biocontrol bacterium which showed a remarkable level of inhibitory activities against several foliar and root pathogens of tea [89]. Due to no hemolysin production, the authors concluded that *S. marcescens* ETR17 can be applied to minimize the use of chemical fungicides for disease control in tea gardens [89]. Woodhams et al. [95] reported that *S. marcescens* PG caused remarkable growth inhibition of chytrid fungi *Batrachochytrium dendrobatidis* and *B. salamandrivorans* at minimal inhibitory concentrations of 10 and 50 μM, respectively [95]. *Streptomyces* fusant NRCF69 PGs have also exhibited antimycotic activity against clinical dermatophyte isolates (*Trichophyton*, *Microsporum*, and *Epidermophyton*) [91].

The PG produced by *S. marcescens* PP1 showed higher inhibitory activity against Gram-positive bacteria (*Staphylococcus aureus* and *Bacillus subtilis*) than Gram-negative bacteria (*E. coli*, *Pseudomonas aeruginosa*, and *Klebsiella pneumoniae*) [82]. Arivizhivendhan et al. reported the inhibitory activity of *S. marcescens* produced against *P. aeruginosa* [93]. Similar results of antibacterial activities by *S. marcescens* 2170 PG have also been reported by Herráez et al. [94]. PG produced by *S. marcescens* UFPEDA 398 showed inhibitory activity against 20 strains of oxacillin-resistant *S. aureus*. The minimum inhibitory concentrations and minimum bactericidal concentrations ranged from 1–4 μg/mL and 2–16 μg/mL, respectively [83].

Arivizhivendhan et al. also reported that the PG from *S. marcescens* showed effective antioxidant and antibacterial activities [92]. The extracts of aloe leaf and cucumber fruit are known to have photoprotective activity. The application of PG as antibacterial and antioxidant additives to the extracts of aloe leaf and cucumber fruit has been evaluated for the potential of developing commercial sunscreens for human skin protection [96]. Nakashima et al. reported that a bacterial strain (MS-02-063) that produces large amounts of PGs showed equivalent antibacterial activity to those of tetracycline against some pathogenic Gram-positive bacteria including *S. aureus* [99]. *Pseudoalteromonas rubra* synthesizes PG, cycloprodigiosin, and four PG derivatives that differ in the length of the alkyl chain. The antimicrobial activities of the produced PG, cycloprodigiosin, and 2-methyl-3-hexyl-prodiginine have been examined against *E. coli*, *Staphylococcus aureus*, *Salmonella typhi*, and *Candida albicans* and it was found that cycloprodigiosin potently inhibited *S. aureus* at a concentration of 20 μg/mL [100].

The antimicrobial effect of PG against microorganisms including antibiotic-resistant pathogens and phytofungal pathogens may provide a potential platform for their use as microbial disinfectants in the fields of pharmaceutical and agricultural biocontrol, respectively.

### 3.2. Antiparasitic Activity

The isolation of antiparasitic compounds from microbial extracts has been widely investigated. The antiparasitic activities of *S. marcescens* produced PG have been evaluated against *Plasmodium falciparum* and *Trypanosoma brucei gambiense*. Combinations of PG with phytosynthesized silver and gold nanoparticles showed a remarkable decrease in the IC_50_ values on both parasites (2.7- to 3.6-fold) without an increase in cytotoxicity to the mammalian cells [102]. The antagonist effect of *S. marcescens* 2170 produced PG has been tested against parasite *Trypanosoma cruzi* cells. The results showed that the concentration of PG required to suppress *T. cruzi* growth was significantly lower than that required for benznidazole (0.25 mg/L and 4.9 mg/L, respectively) [94]. Isaka et al. reported three compounds (metacycloprodigiosin, bafilomycin A_1_, and spectinabilin) extracted from *Streptomyces spectabilis* BCC 4785 possessed potent antimalarial activity against *P. falciparum* K1. Among them, metacycloprodigiosin exhibited potent inhibitory activity (IC_50_ of 0.0050 μg/mL) against *P. falciparum* K1 with much lower cytotoxicity [105]. Marchal et al. observed that the presence of a nitrogen atom in the A-ring of PGs is needed for antimalarial activity [107]. Later, the antimalarial activities of four natural and three synthetic PGs were estimated against *P. falciparum* [108]. From a culture of α-proteobacteria, Lazaro et al. purified heptyl prodigiosin which showed antimalarial activity similar to that of quinine against the chloroquine-sensitive strain *P. falciparum* 3D7 [109].

The lack of useful nematicides and the serious damage caused by plant-parasitic nematodes have led to an urgent requirement to isolate some natural remedy for their control [102]. The antinematicidal activity of *S. marcescens* produced PG has been studied against juvenile stages of *Radopholus similis* and *Meloidogyne javanica*. The results showed better activity of this PG (LC_50_ values, 83 and 79 μg/mL, respectively) than those of the positive control of copper sulfate (LC_50_ values, 380 and 280 μg/mL, respectively). The PG also showed antagonist effect on nematode egg-hatching ability [102].

### 3.3. Insecticidal Activity

Bioagents produced from microbes offer alternatives to chemical pesticides as they can be more selective and safer than chemical insecticides. The insecticidal potency of *S. marcescens* TKU011 PG was investigated and compared with those of food colorants Tartrazine (Y4) and Allura Red AC (R40) against *Drosophila* larvae [60,61]. The LC_50_ of PG (0.23 µg/µL), Y4 (0.449 µg/µL), and R40 (30 µg/µL) using a five-day exposure period showed potential toxicity of the biopigment PG and the food colorant Y4 against *Drosophila* larvae [60]. The order of survival rates of *Drosophila* larvae after five-day feeding with 30 µL of cell broth, broken cell, cell pellet, or cell-free culture supernatant of *S. marcescens* TKU011 were 0%, 6.25%, 12.5%, and 37.5%, respectively, indicating a direct relationship with their PG content. The fractions with a high PG concentration also exhibited a high insecticidal activity [61].

Patil et al. reported the mosquito larvicidal potential of the crude PG extracted from *S. marcescens* NMCC46 culture broth against *Aedes aegypti* and *Anopheles stephensi*. The LC_50_ values of second, third, and fourth instars of *A. aegypti* were 41.65, 139.51, and 103.95 and those of *A. stephensi* were 51.12, 105.52, and 133.07 [110]. Pure PG may be an important molecule for the control of *Aedes aegypti* and *Anopheles stephensi* mosquitoes at their larval and pupal stages [111]. Suryawanshi et al. [111] analyzed the effect of the pure PG isolated from *S. marcescens* NMCC 75 against larval and pupal stages of *Aedes aegypti* and *Anopheles stephensi* mosquitoes. The pure PG showed mosquito larvicidal activities with LC_50_ values of 14, 15.6, 18, and 21 µg/mL against the early second, third, fourth instars, and pupal stages of *Aedes aegypti*, respectively, and with LC_50_ values of 19.7, 24.7, 26.6, and 32.2 µg/mL, respectively, against the early second, third, and fourth instars and pupae of *Anopheles stephensi*, respectively. The results of variation of enzymes (esterases, acetylcholine esterases, phosphatases, and proteases) and total proteins in the fourth instar larvae of *Aedes aegypti* indicated the intrinsic difference in biochemical features as a result of PG treatment [111]. Zhou et al. observed PG produced by *S. marcescens* SCQ1 (an entomopathogenic strain isolated from silkworm) causes acute septicemia in silkworms [112]. Asano et al. investigated the synergistic effects of PG, chitinases, and chitin-binding protein from *S. marcescens* ATCC274 on the insecticidal activity of δ-endotoxin (*Cry* 1C of *Bacillus thuringiensis*) against the common cutworm, *Spodoptera litura* [113]. The results showed that only PG exhibited a remarkable synergistic inhibitory activity with *Cry* 1C which was lethal and growth inhibitory. The supernatants from *S. marcescens* containing PG and partially purified PG exhibited a similar synergistic activity on the insecticidal activity of *Cry*1C [113].

### 3.4. Anti-Cancer Activity

Several studies have demonstrated the anti-cancer activity of PG in various types of cancer. Zhou et al. reported the PG produced by *S. marcescens* SCQ1 (an entomopathogenic strain) showed potent anticancer activity on human lung adenocarcinoma A549 cells and concluded that *S. marcescens* SCQ1 may have potential to be used as an anti-cancer compound [112]. To study the role of mitochondria in PG-induced apoptosis, Llagostera et al. evaluated the apoptotic action of PG in GLC4 small cell lung cancer cell line by Hoechst 33342 staining and found that PG induces apoptosis in both caspase-dependent and caspase-independent pathways [119]. The high cytotoxic sensitivity of the human small cell lung doxorubicin-resistant carcinoma (GLC4/ADR) cell line to PG through apoptosis activation was further studied and concluded that the results support PG as a potential drug for the treatment of lung cancer because of its ability to overcome the multidrug resistance phenotype produced by MRP-1 overexpression [120]. The synergistic effects between PG (produced by *Vibrio* sp. C1-TDSG02-1) and doxorubicin (a chemotherapy drug) against oral squamous cell carcinoma (OSCC) cells was reported by Lin and Wen [118]. Based on the results of PG-priming, autophagy could sensitize OSCC cells by promoting doxorubicin influx without regulation of doxorubicin transporter. Lin and Wen also concluded that the PG (produced by *Vibrio* sp. C1-TDSG02-1)-priming might be a promising adjuvant approach for the chemotherapy of OSCC [118]. Chiu et al. reported the attenuation of tumors accumulated in the mice trachea by PG (produced by *Vibrio* sp. C1-TDSG02-1) treatment and concluded the potential of PG as a potential chemotherapeutic agent for lung cancer regimens regardless of doxorubicin resistance [116].

Montaner and Perez-Tomas evaluated the apoptotic action of PG in colon cancer cells (DLD-1 and SW-620 human colon adenocarcinoma cells, NRK, and Swiss-3T3 nonmalignant cells) and found that metastatic SW-620 cells were more sensitive to PG than DLD-1. According to the results in both cancer cell lines, the authors suggested that PG induces apoptosis in colon cancer cells [130]. The effect of PG on proliferation and expression of apoptotic-related genes in HT-29 cells was evaluated by Hassankhani et al., who also suggested that PG-induced apoptosis may ascribe to the inhibition of Bcl-2 and survivin in HT-29 cells and these genes may be promising molecular targets of PG [128]. Dalili et al. [129] evaluated the antiproliferative activities of *S. marcescens* PTCC 1111 produced PG in HT-29 and T47D cancer cell lines. The results showed that HT-29 cells were more sensitive than T47D cells to PG [129]. PG showed higher apoptotic effect than doxorubicin in HT-29 cells. The authors, therefore, suggested the use of PG as a promising antineoplastic agent that triggers apoptosis in different cancer cell lines [129]. Kavitha et al. indicated strong anti-cancer and apoptotic activity of *S. marcescens* PG against human cervical carcinoma cancer, according to the results of dose-dependent inhibition of human cervical carcinoma cell (Hela-229 cell line) proliferation [137]. Díaz-Ruiz et al. found that treatment of human gastric carcinoma cells (HGT-1 cell line) with PG showed a constant decrease in viability due to apoptosis and suggested that PG induces apoptosis in HGT-1 cells [133].

Soto-Cerrato et al. observed potently cytotoxic activity of PG in both estrogen receptor-positive (MCF-7) and negative (MDA-MB-231) breast cancer cell lines and suggested PG as an interesting and potent new pro-apoptotic agent for the treatment of breast cancer despite the presence of multidrug resistance transporter molecules [122,123]. Lu et al. reported that PG could downregulate RAD51 (an attractive target for anticancer drugs) in multiple human breast carcinoma cell lines irrespective of p53 status [126].

To evaluate the immunosuppressive and apoptotic mechanisms of PG, Monge et al. examined the variation of protein expression on exposure to apoptotic concentrations of PG in mitoxantrone (MCF-7-MR) resistant MCF-7 cancer cell line and found that the identified proteins were involved in various cellular functions, including cell defense, DNA repair, and cellular organization [121]. Sam and Pourpak reported that as molecular targets of PG, P53 and survivin contribute to caspase-3-dependent apoptosis in acute lymphoblastic leukemia cells where PG represents an attractive p53- and survivin-modulating agent [134]. Campàs et al. demonstrated that PG induces apoptosis of B-cell chronic lymphocytic leukemia (B-CLL) cells through caspase activation [114]. Liu et al. evaluated the undecylprodigiosin (UPG) extracted from the fermentation broth of *Saccharopolyspora* sp. (a sponge Mycale plumose-derived actinomycete) and found a significant cytotoxic activity of UPG against five cancer cell lines, especially on murine leukemia P388 [115]. Liu et al. further indicated that UP inhibits proliferation of P388 by inducing G2/M phase arrest and apoptosis, which was related to the activation of P38, JNK rather than ERK1/2 signaling [214]. Based on the observation that PG significantly increased the rate of growth inhibition and decreased metabolic activity of HepG2 cells in a dose- and time-dependent manner, Yenkejeh et al. suggested PG as an attractive compound that may provide a novel approach to the hepatocellular carcinoma-targeted therapy [135].

Based on the results of dose-dependent inhibition of human cervix carcinoma cell (Hela-229 cell line) proliferation by *S. marcescens*, Kavitha et al. suggested strong anticancer and apoptosis activity of PG against human cervical carcinoma cancer [137]. Montaner et al. [142] studied the effects of *S. marcescens* 2170 culture supernatant on the viability of different hematopoietic cancer cell lines (Jurkat, NSO, HL-60, and Ramos) and nonmalignant cells (NIH-3T3 and MDCK) and found that the cytotoxic effect was due to apoptosis [142]. The results of using mutants of *S. marcescens* (strains OF, WF, and 933) that do not synthesize PG suggested the involvement of PG in this apoptosis [142,143]. Li et al. [143] investigated the anticancer activities and mechanism of activity of *S. marcescens* HDZK-BYSB107 PG by using human choriocarcinoma (JEG3) and prostate cancer cell lines (PC3) in vitro and JEG3 and PC3 tumor-bearing nude mice in vivo [143]. The bacterial PG was observed to induce apoptosis in JEG3 cells, and PG significantly inhibited the growth of JEG3 and PC3 cells, in a dose and time-dependent manner [143]. Nguyen et al. [59] found that the PG purified from fermentation of chitin-containing medium by *S. marcescens* TKU011 showed potent anticancer activities against A549, Hep G2, MCF-7, and WiDr with IC_50_ values of 0.06, 0.04, 0.04, and 0.2 µg/mL, respectively. For comparison, mitomycin C, a commercial anti-cancer compound was also tested, and it showed weaker activity with IC_50_ values of 0.11, 0.1, 0.14, and 0.15 µg/mL, respectively [59]. Muthukumar et al. [145] studied the antioxidant, anti-inflammatory, and cytotoxic properties of PG produced by *S. marcescens* VITAPI and observed potent radical scavenging effect of the extracted PG at 86%, which was significant in comparison to ascorbic acid as a standard [145]. The in vitro anti-inflammatory effect of PG in controlled experimental conditions revealed its protection at 88% and inhibition in a concentration-dependent manner. The cytotoxic bioassay of PG showed the IC_50_ value as 50 μg/mL at 63% cytotoxicity [145]. El-Bondkly et al. [91] reported the cytotoxic activities of PG pigments produced by *Streptomyces* fusant NRCF69 against three human cancer cell lines, including colon cancer cell line (HCT-116), liver cancer cell line (HEPG-2), and breast cancer cell line (MCF-7) [91]. The synthesis and evaluation of a series of ten PGs that bear ester and amide substitution about the C-ring of the PG skeleton were reported by Lund et al. [136]. The PG bearing C-ring esters and amides obtained by chemical synthesis displayed anticancer activity, particularly when featuring a hexyl chain [136].

### 3.5. Algicidal Activity

Red tides (harmful algal blooms) are caused by some toxic phytoplankton, and lead to massive economic losses and cause marine environmental disturbances. The PG producing strain *Hahella chejuensis* KCTC 2396 (a marine bacterium) was evaluated by Kim et al. as an effective and environment-friendly strategy to control red tide outbreaks [148]. The lytic activity of this promising molecule against *Cochlodinium polykrikoides* cells at very low concentrations (1 ppb) was serendipitously detected, making *H. chejuensis* a strong candidate among the biological agents to control red tides [148]. Nakashima et al. [149] reported that the pigment, PG-L-1 produced by a marine bacterium strain MS-02-063 (γ-proteobacterium) exhibited potent algicidal activity against various red tide phytoplanktons in a concentration-dependent manner [99,149]. The authors suggested that PG-L-1 produced by strain MS-02-063 is controlled by the homoserine lactone quorum sensing. This bacterium and other algicidal bacteria may be effective in regulating the blooms of harmful flagellate algae through the quorum sensing system [149].

### 3.6. Dyes

The environmental pollution concerns raised because of the use of chemically synthetic pigment have led to increasing interest in the natural ones. Microbial pigments have lately attracted increasing attention in textile dyeing because of their sustainability and cleaner production [75,76,77,78,151,152,169,210]. Recently, PG has become a research hotspot for its bright colors and antibacterial function. To promote the application of PG in textile dyeing, a novel idea of preparing dye liquid based on fermentation broth was put forward via increasing the proportion of extracellular pigments [151]. With the improvement in transmembrane transfer efficiency of *S. marcescens* ATCC8100, PG was produced as the proportion of extracellular pigments, and the complicated biological separation process could be avoided and the application of microbial pigments in textile dyeing can be promoted [151].

In the ethanol solution, PG has good stability under natural indoor light but gets rapidly decomposed under intense sunlight [210]. PG is an eco-friendly colorant to dye fabrics, including synthetic and natural fibers. Synthetic fabrics (such as polyamide and acrylic) dyed with PG have high colorfastness to washing and exhibit antimicrobial activities against *E. coli* and *Staphylococcus aureus*. Liu et al. [210] reported the promising prospects of *S. marcescens* jx1-1, with high PG yield and purity, in food, cosmetic, and textile industries.

A strain of *Vibrio* sp. KSJ45 produced large quantities of bright red PGs with an elementary composition of C_20_H_25_N_3_O that could be used to dye many fibers including wool, nylon, acrylics, and silk. Fabrics dyed with the PGs produced from *Vibrio* sp. KSJ45 demonstrated antibacterial activity [101]. Vaidyanathan et al. [211] have studied the application of a novel red biochrome (514 Da in size) produced by solid-state cultivation of *Serratia sakuensis* subsp. nov. strain KRED in the dyeing of silk, wool, and cotton fabrics. The results showed that silk, wool, and cotton fabrics dyed with this new natural red compound have high color strength and dye uptake values along with good colorfastness as well as antibacterial activity [211]. Mehta and Shah [169] studied the application of PG in the candle industry. The mixture of PG with translucent wax was homogenized and poured into the mold and the candles were left to cool down. After de-molding, the translucent candle showed a more intense coloration, which was similar to the colored candles available in the market, the authors proposed that the synthetic coloring agents can be replaced by natural colorants extracted from microorganisms. The results of the present study on the isolation and application of PG extracted from *S. marcescens* in the coloration of translucent candles revealed that the pigment PG can be considered as a possible alternate source of colorant in various industries. Table 2 summarizes the bioactivities and applications of PGs reported.

## 4. Conclusions and Perspectives

Pigments of various kinds and forms have been used as additives or supplements in the food industry, cosmetics, pharmaceuticals, biocontrol, and other applications. Recently, in response to the problems of the synthetic pigments that cause toxicity and carcinogenicity in the human body, the inclination to use natural pigments as adding natural materials for human health and safety has gradually expanded. This review contains the most recent information on the production of PGs from various bacteria especially *S. marcescens*. For PG production, the commercial media of peptone and yeast extract supplemented with vegetable oil have been used for enhancing PG productivity. Considering the bioactivities of PG, anti-cancer applications have been most widely studied. Compared to the use of expensive media supplemented with vegetable oils, chitin and protein-containing fishery byproducts have more potential for PG production.

The discovery of inexpensive PG not only solves environmental problems but also promotes the economic value of marine wastes. Furthermore, the chitinolytic and proteolytic enzymes together with PG produced by *S. marcescens* TKU011 using fishery byproducts can enhance antimicrobial activities and may be a potential source of biological control agents.

## Figures and Tables

**Table 1:** Comparison of prodigiosin (PG) yield by *S. marcescens* in different reports.

Culture Medium	PG	Cultivation	Reference
(C/N Source)	Productivity (mg/L)	Temp/pH/Time	
Chitin/casein	4620	30 °C 1 Day/25 °C 2 Day	[59]
Squid pens	2480		[61]
Shrimp shells	190		[61]
Shrimp heads	30		[61]
Crab shells	110		[61]
Ram horn peptone/mannitol	277.74	48 h	[170]
Peanut seed broth	47,000	-	[91]
Peanut seed broth	3875	28 °C/-/36 h	[201]
Peanut seed broth	1595	28 °C after 48 h	[82]
Peanut oil	2890		[201]
Peanut powder/olive oil/beef extract	13,622		[212]
Sunflower seed broth/maltose	1556		[82]
Sunflower seed broth/glucose	1525		[82]
Sesame seed broth	16,680	28 °C/-/36 h	[201]
Sesame oil	1006		[201]
Coconut oil	1420		[201]
Copra seed	1940		[201]
Casein-enriched broth/vegetable oil	765.1	30 °C/7/84 h	[81]
Ethanol/cottonseed meal	3000	28 °C/6.8/72 h	[190]
Casein	4280		[206]
Cassava wastewater/mannitol	49,500	28 °C/7/48 h	[174]
Yeast extract	690		[176]
Yeast extract	380.2		[212]
Yeast/tryptone	152	30 °C/72 h	[172]
Nutrient broth/glycerol		25 °C/72 h	[169]
Tryptone	353		[212]
Soya peptone	1174		[212]
Peptone/maltose	656	28 °C/72 h	[143]
Sweet potato powder/casein	4800		[171]
Soybean powder	0		[212]
Corn steep liquor	0		[212]
Modified Luria–Bertani broth/sunflower oil	790		[175]
3-[*N*-morpholino]-Ethanesulfonic acid	475		[199]

**Table 2:** Bioactivities and applications of PGs.

Strain	Bioactivity/Application	Reference
(Antimicrobial activity)		
*S. marcescens* UFPEDA398	antibacterial	[83]
*S. marcescens* IBRL USM 84	antibacterial	[84]
*S. marcescens*	antibacterial	[94]
*S. marcescens*	antibacterial	[92,93]
*S. marcescens*	antibacterial	[97]
*S. marcescens*	anti-chytrid fungi	[95]
*S. marcescens* PP1	anti-fungal pathogens, antibacterial	[82]
*S. marcescens* CFFSUR-B2	anti-fungal pathogens	[88]
*S. marcescens* ETR17	anti-fungal pathogens	[89]
*S. marcescens*	antibacterial	[96]
*S. marcescens* SR_1_	anti-fungal pathogens	[81]
*S. marcescens* B2	anti-fungal pathogens	[85,86]
*Streptomyces* fusant NRCF69	antidermatophytic	[91]
Strain MS-02-063	antibacterial	[99]
*Pseudoalteromonas rubra*	antibacterial	[100]
*Vibrio* sp. KSJ45	antibacterial	[101]
(Anti-parasitic activity)		
*S. marcescens* 2170	anti-parasitic euglenoids	[94]
*S. marcescens*	anti-parasitic euglenoids	[102]
*S. marcescens*	anti-nematode	[103]
*S. marcescens*	anti-malaria	[104]
*Streptomyces spectabilis* BCC 4785	anti-malaria	[105]
*Streptomyces* sp.	anti-malaria	[106]
Prodigiosene	anti-malaria	[107]
Prodiginine	anti-malaria	[108]
Heptyl prodigiosin	anti-malaria	[109]
(Insecticidal activity)		
*S. marcescens* TKU011	fruit fly larvicide	[60,61]
*S. marcescens* NMCC46	mosquito larvicide	[110]
*S. marcescens* NMCC75	mosquito larvicide	[111]
*S. marcescens* SCQ1	acute septicemia of silkworm	[112]
*S. marcescens* ATCC274	anti-cutworm	[113]
(Anti-cancer activity)		
*S. marcescens* TKU011 *S. marcescens* TNU01	anti-cancer anti-cancer	[59] [87]
*S. marcescens*	anti-cancer	[138]
*S. marcescens*	anti-cancer	[147]
*S. marcescens* SCQ1	anti-lung cancer	[112]
*Vibrio* sp. C1-TDSG02-1	anti-lung cancer	[116]
*S. marcescens*	anti-lung cancer	[119,120]
*S. marcescens*	anti-oral cancer	[117]
*Vibrio* sp. C1-TDSG02-1	anti-oral cancer	[118]
*S. marcescens*	anti-breast cancer	[97]
*S. marcescens*	anti-breast cancer	[121]
*S. marcescens*	anti-breast cancer	[124]
*S. marcescens*	anti-breast cancer	[125]
*S. marcescens*	anti-breast cancer	[126]
*S. marcescens*	anti-breast cancer	[127]
*S. marcescens*	anti-breast cancer	[122,123]
*S. marcescens*	anti-colorectal cancer	[128]
*S. marcescens*	anti-colorectal cancer	[129]
*S. marcescens*	anti-colorectal cancer	[130]
*S. marcescens*	anti-colorectal cancer	[131]
*S. marcescens*	anti-colorectal cancer	[132]
*S. marcescens*	anti-gastric cancer	[133]
*S. marcescens*	anti-leukemia	[114]
*S. marcescens*	anti-leukemia	[134]
*Saccharopolyspora* sp.	anti-leukemia	[214]
*S. marcescens*	anti-hepatocellular cancer	[135]
Prodigiosene	anti-cancer	[136]
Prodigiosin	anti-cancer	[141]
*S. marcescens*	anti-cervix carcinoma	[137]
*S. marcescens* MTCC97	anti-cervix carcinoma	[140]
*S. marcescens* 2170	anti-hematopoietic cancer	[142]
*S. marcescens* HDZK-BYSB107	anti-choriocarcinoma	[143]
*S. marcescens* HDZK-BYSB107	anti-prostate cancer	[143]
*Streptomyces fusant* NRCF69	anti-colon cancer	[91]
*S. fusant* NRCF69	anti-liver cancer	[91]
*S. fusant* NRCF69	anti-breast cancer	[91]
(Anti-oxidation/Anti-inflammatory activity)		
*S. marcescens* VITAPI	anti-oxidation	[145]
*S. marcescens* VITAPI	anti-inflammatory	[145]
*S. marcescens*	immunosuppressive	[147]
(Algicidal activity)		
*Hahella chejuensis* KCTC 239	algicide	[148]
strain MS-02-063 (γ-proteobacterium)	algicide	[149]
(Dyes)		
*S. marcescens* ATCC8100	textile	[151]
*Serratia* sp. KH-1	textile	[152]
*S. marcescens*	candle	[163]
*S. marcescens*	sunscreen	[96]
*Serratia rubidaea*	textile	[154,177]
*Vibrio* sp. KSJ45	textile	[101]
